# Dexmedetomidine suppresses glucose‐stimulated insulin secretion in pancreatic β‐cells

**DOI:** 10.1002/2211-5463.13960

**Published:** 2024-12-20

**Authors:** Munenori Kusunoki, Kiichi Hirota, Tomohiro Shoji, Takeo Uba, Yoshiyuki Matsuo, Mikio Hayashi

**Affiliations:** ^1^ Department of Anesthesiology Kansai Medical University Hirakata Osaka Japan; ^2^ Department of Pain Clinic Medicine Minamiuonuma City Hospital Minamiuonuma Niigata Japan; ^3^ Department of Anesthesiology Mitoyo General Hospital Kan‐onji Kagawa Japan; ^4^ Department of Human Stress Response Science Institute of Biomedical Science, Kansai Medical University Hirakata Osaka Japan; ^5^ Department of Cell Physiology Institute of Biomedical Science, Kansai Medical University Hirakata Osaka Japan

**Keywords:** dexmedetomidine, insulin secretion, K_ATP_ channel, patch clamp

## Abstract

Proper glycemic control is crucial for patient management in critical care, including perioperative care, and can influence patient prognosis. Blood glucose concentration determines insulin secretion and sensitivity and affects the intricate balance between the glucose metabolism. Human and other animal studies have demonstrated that perioperative drugs, including volatile anesthetics and intravenous anesthetics, affect glucose‐stimulated insulin secretion (GSIS). Dexmedetomidine (DEX) decreases insulin release and affects glucose metabolism; however, the specific mechanism underlying this phenomenon remains largely unknown. Thus, we investigated the effect and mechanism of DEX on insulin secretion using mouse and rat pancreatic β‐cell‐derived MIN6 and INS‐1 cell lines and primary pancreatic β‐cells/islets extracted from mice. The amount of insulin secreted into the culture medium was determined using an enzyme‐linked immunosorbent assay. Cell viability, cytotoxicity, and electrophysiological effects were investigated. Clinically relevant doses of DEX suppressed GSIS in MIN6 cells, INS‐1 cells, and pancreatic β‐cells/islets. Furthermore, DEX suppressed insulin secretion facilitated by insulinotropic factors. There was no significant difference in oxygen consumption rate, intracellular ATP levels, or caspase‐3/7 activity. Electrophysiological evaluation using the patch‐clamp method showed that DEX did not affect ATP‐sensitive potassium (K_ATP_) channels, voltage‐dependent potassium channels, or voltage‐gated calcium channels. We demonstrated that clinically relevant doses of DEX significantly suppressed GSIS. These findings suggest that DEX inhibits a signaling pathway via α2‐adrenoceptor or insulin vesicle exocytosis, resulting in GSIS suppression. Our results support the hypothesis that DEX suppresses insulin secretion and reveal some underlying mechanisms.

## Graphical abstract

Dexmedetomidine decreases insulin release and affects glucose metabolism; however, the specific mechanism remains largely unknown. This study shows clinically relevant doses of dexmedetomidine suppressed glucose‐stimulated insulin secretion. There was no significant difference in oxygen consumption rate, intracellular ATP levels, or caspase‐3/7 activity. Electrophysiological evaluation showed that dexmedetomidine did not affect ATP‐sensitive potassium channels, voltage‐dependent potassium channels, or voltage‐gated calcium channels.

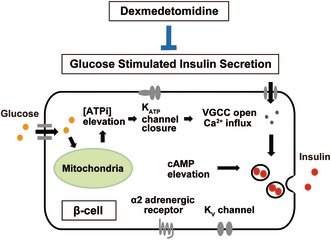

**Abbreviations**
ANOVA: analysis of variance
BAPTA: 1,2‐bis(2‐aminophenoxy)ethane‐*N,N,N*′*N*′‐tetraacetic acid
DEX: dexmedetomidine
ECAR: extracellular acidification rate
FCCP: carbonyl cyanide‐p‐trifluoromethoxyphenylhydrazone
GSIS: glucose‐stimulated insulin secretion
OCR: oxygen consumption rate
SD: standard deviation

In critical care settings, including perioperative care, proper glycemic control is crucial for patient management [1, 2]. Hyperglycemia is a major risk factor for morbidity and mortality in patients requiring critical care [1]. Blood glucose concentration determines insulin secretion and sensitivity and affects the intricate balance between the glucose metabolism. On the other hand, external factors such as stress from surgical procedures and drugs administered for anesthetic management can substantially affect glycemic regulation [2].

Human and other animal studies have demonstrated that perioperative drugs including volatile anesthetics have an effect on glucose‐stimulated insulin secretion (GSIS) [3, 4, 5]. Suzuki *et al*. [4] demonstrated the molecular mechanisms underlying the suppression of insulin secretion by volatile anesthetics. Additionally, our previous study indicated that intravenous anesthetic propofol enhances insulin secretion in pancreatic β‐cells [6]. Dexmedetomidine (DEX), a novel α2 adrenergic agonist with sedative, analgesic, anti‐inflammatory, and organ‐protective effects, is widely used in anesthesia and intensive care [7]. It has been demonstrated that DEX decrease insulin release and affects glucose metabolism [8, 9]; however, the specific mechanisms underlying this phenomenon remain unknown. In this study, we investigated the effect of DEX on insulin secretion in response to high glucose levels using cell biological and electrophysiological methods.

## Methods

### Reagents

DEX and 8‐Br‐cAMP were obtained from Sigma‐Aldrich (St. Louis, MO, USA). Glibenclamide was obtained from Wako Pure Chemical Industries, Ltd. (Osaka, Japan) and diazoxide was procured from Abcam (Cambridge, MA, USA). Stromatoxin‐1 was purchased from Alomone Labs Ltd. (Jerusalem, Israel).

### Cells and cell culture

Mouse insulinoma MIN6 cells were gifted by Dr. J Miyazaki (Osaka University) [10]. MIN6 cells were cultured in Dulbecco's modified Eagle's medium (Gibco, Grand Island, NY, USA) containing 450 mg·dL^−1^ glucose, 10% fetal bovine serum (FBS), 50 μm β‐mercaptoethanol, 100 U·mL^−1^ penicillin, and 0.1 mg·mL^−1^ streptomycin [11]. Rat INS‐1 cells were cultured in RPMI1640 (Sigma‐Aldrich) supplemented with 10% FBS, 10 mm HEPES, 2 mm l‐glutamine, 1 mm sodium pyruvate, 50 μm β‐mercaptoethanol, 100 U·mL^−1^ penicillin, and 0.1 mg·mL^−1^ streptomycin [12].

### Isolation of mouse pancreatic islets

All animal experiments were approved by the Animal Experimentation Committee of the Kansai Medical University (Approval number: 21‐087) and performed in accordance with the relevant guidelines and regulations. All methods are reported in accordance with the ARRIVE guidelines. Male *C57BL/6JJcl* mice (8–10 weeks old, *n* = 6) were killed by cervical dislocation. Pancreatic islets were isolated using enzymatic digestion of the pancreas as previously described [13], with slight modifications. Briefly, the pancreas was removed and digested with collagenase (Type IV, 195 U·mL^−1^; Worthington Biochemical, Lakewood, NJ, USA) in a solution containing 2 mm glucose and trypsin inhibitor (0.01%; Sigma‐Aldrich) at 37 °C for 30 min with vigorous shaking [6]. The pancreatic tissue was triturated using a pipette and washed twice with an enzyme‐free solution. The islets were selected using a glass micropipette under a stereomicroscope. Batches of 10 islets were used to measure the insulin concentration.

### Measurement of insulin concentration

Insulin concentration in the culture medium of MIN6 cells, INS‐1 cells, and pancreatic β‐cells/islets isolated from mice was assessed using the Mouse/Rat Insulin H‐type enzyme‐linked immunosorbent assay kit (Shibayagi Co. Ltd., Shibukawa, Japan) following the manufacturer's instructions [4]. In brief, MIN6 cells were cultured for 30 min in Krebs‐Ringer bicarbonate HEPES (KRBH) buffer (140 mm NaCl, 3.6 mm KCl, 0.5 mm NaH_2_PO_4_, 0.5 mm MgSO_4_, 1.5 mm CaCl_2_, 2 mm NaHCO_3_, 10 mm HEPES, and 0.1% bovine serum albumin [BSA]) containing 2 mm glucose [6]. The buffer was then changed to another KRBH buffer solution containing glucose at the relevant experimental concentration, and the cells were cultured for 1 h. The KRBH buffer was collected, and its insulin concentration was measured. Our previous study indicated that the compensation of total protein weight did not impact insulin concentration measurement [6]. Thus, we quantified insulin concentration without compensation in the case of MIN6 and INS‐1 cells. The results were normalized to the concentration of control samples of each independent experiment, and the normalized values were shown as insulin secretion ratio.

### Insulin content assay

Whole‐cell lysates were prepared by incubating cells for 15 min in cold radioimmune precipitation assay buffer with Complete Protease Inhibitor Cocktail Tablets (Roche Diagnostics, Tokyo, Japan) after twice washing with PBS. Samples were then centrifuged at 10000 × g to allow the cell debris to settle to obtain total cell lysates. Then, the insulin content of the cell lysate was assayed using the Mouse/Rat Insulin H‐type enzyme‐linked immunosorbent assay kit (Shibayagi Co. Ltd., Shibukawa, Japan). The insulin content was compensated with total protein weight.

### Caspase activity assay

The activities of caspase‐3 and caspase‐7 were determined using the Caspase‐Glo 3/7 3D Assay Kit (Promega, Madison, WI, USA) according to the manufacturer's protocol [14, 15]. Briefly, cells were seeded at a density of 2 × 10^4^ cells·well^−1^ in 96‐well plates and incubated overnight. The cells were then treated with camptothecin and DEX for varying periods. After treatment, Caspase‐Glo 3/7 3D Assay reagent was added to each well. The cells were incubated at room temperature for 30 min, and the luminescence of each well was measured using the EnSpire Multimode Plate Reader (PerkinElmer, Waltham, MA, USA). Caspase activity was then calculated by comparing the levels of luminescence of the treated cells with those of the control cells (incubated without drugs), with the latter defined as 100%.

### ATP assay

The intracellular ATP level was evaluated using the CellTiter‐Glo 2.0 cell viability assay kit (Promega) [16]. Briefly, cells were seeded at a density of 2 × 10^4^ cells·well^−1^ in 96‐well plates and allowed to grow for 1 h in the presence or absence of DEX [6]. CellTiter‐Glo 2.0 reagent was then added directly into each well, and the samples were incubated for 10 min before reading using an EnSpire Multimode Plate Reader (PerkinElmer). This detected the luminescence generated by the luciferase‐catalyzed reaction between luciferin and ATP. The ATP level was then calculated by comparing the luminescence levels of cells with those of cells incubated with 2 mm glucose without DEX, defined as 100%.

### Measurement of cellular oxygen consumption and extracellular acidification

Cellular oxygen consumption rate (OCR) and extracellular acidification rate (ECAR) were measured using an XFp extracellular flux analyzer (Agilent Technologies, Santa Clara, CA, USA) [16]. MIN6 cells were seeded at a density of 1 × 10^4^ cells·well^−1^ in an XFp cell culture microplate. The XF Cell Mito Stress Test™ for OCR was assessed in glucose‐containing XF base medium, according to the manufacturer's protocol. The sensor cartridge of the XFp Analyzer was hydrated at 37 °C in a non‐CO_2_ incubator 1 day before the experiment. For the OCR assay, injection port A on the sensor cartridge was loaded with oligomycin (a complex V inhibitor, final concentration 1 μm), port B was loaded with carbonyl cyanide‐p‐trifluoromethoxyphenylhydrazone (FCCP; an uncoupling agent, final concentration 2 μm), and port C was loaded with rotenone/antimycin A (inhibitors of complexes I and III, respectively, final concentration 0.5 μm each). During sensor calibration, the cells were incubated at 37 °C in a non‐CO_2_ incubator in 180 μL assay medium (XF Base Medium: 25 mm glucose, 1 mm pyruvate, and 2 mm l‐glutamine, pH 7.4). The plate was immediately placed on the calibrated XFp Analyzer for the assay.

### Electrophysiological studies

MIN6 cells were incubated in an extracellular bath solution containing 2 mm glucose at 37 °C for 30 min before patch‐clamp experiments [6, 17, 18]. The extracellular solution consisted of Krebs‐Ringer bicarbonate HEPES (KRBH) buffer (140 mm NaCl, 3.6 mm KCl, 0.5 mm NaH_2_PO_4_, 0.5 mm MgSO_4_, 1.5 mm CaCl_2_, 2 mm NaHCO_3_, 10 mm HEPES [pH 7.4 with NaOH], and 0.1% BSA). In gramicidin‐perforated patch experiments, gramicidin D (Sigma‐Aldrich) was dissolved in dimethyl sulfoxide (DMSO) at 20 mg·mL^−1^ and then diluted to a final concentration of 0.1 mg·mL^−1^ in a KCl‐rich pipette solution containing 150 mm KCl and 10 mm HEPES (pH 7.4 with KOH). For whole‐cell recording of voltage‐dependent outward K^+^ currents, the K^+^ pipette solution contained 60 mm potassium aspartate, 65 mm KCl, 1 mm KH_2_PO_4_, 5 mm ethylenediaminetetraacetic acid, 3 mm K_2_ATP, and 5 mm HEPES (pH 7.4). For Ca^2+^ channel current recording, the Cs‐gluconate pipette solution contained 100 mm Cs‐gluconate, 10 mm CsCl, 1 mm MgCl_2_, 5 mm 1,2‐bis(2‐aminophenoxy)ethane‐*N,N,N*′*N*′‐tetraacetic acid (BAPTA), 5 mm ATP‐Mg, 0.3 mm GTP, 10 mm glucose, and 10 mm HEPES (pH 7.4 with CsOH). The extracellular solution consisted of 140 mm NaCl, 3.6 mm KCl, 5 mm BaCl_2_, 0.5 mm MgCl_2_, 10 mm HEPES (pH 7.4 with NaOH), and 0.1% BSA. The patch pipettes (G‐1.5; Narishige, Tokyo, Japan) had a resistance of 5–7 MΩ when filled with pipette solutions. The membrane potential was corrected for the liquid junction potential at the tip of the patch pipette in the KRBH buffer, and the tip of the indifferent reference electrode was filled with the KRBH buffer and placed in the bath. All experiments were conducted at 23–30 °C. Membrane potential measurements and whole‐cell current recordings were performed using an EPC 800 patch‐clamp amplifier (HEKA Elektronik, Inc. Holliston, MA, USA). The amplifier was driven by Clampex 9 (Axon Instruments, Union City, CA, USA) to allow the delivery of a voltage‐step protocol with concomitant digitization of the current. The capacitance transient current was compensated for by the amplifier. Whole‐cell capacitance and series resistance (*R*_s_) were 4.58 ± 0.99 pF and 13.44 ± 1.90 MΩ (*n* = 5), respectively. The *R*_s_ was not electronically compensated during the experiments, and the potentials reported here have not been corrected for *R*_s_. The whole‐cell current was filtered at 1 kHz with an internal four‐pole Bessel filter, sampled at 2 kHz, and transferred to digital signals using Digidata 1322A (Axon Instruments). Subsequent current analysis was performed using Clampfit 9 (Axon Instruments).

### Measurement of intracellular Ca^2+^ concentration

MIN6 cells were grown on glass bottom dishes. Cells were loaded with 3 μm Fura2‐AM (Dojindo, Kumamoto, Japan) in a standard bath solution for 30–45 min in the dark at 37 °C. The cells were perfused with the standard bath solution (115 mm NaCl, 3.6 mm KCl, 0.5 mm NaH_2_PO_4_, 0.5 mm MgSO_4_, 1.5 mm CaCl_2_, 25 mm NaHCO_3_, 10 mm HEPES, and 2 mm glucose). The solution was equilibrated with 5% CO_2_ in O_2_. The temperature was kept at 37 °C. For Ca^2+^‐free solution, 0.5 mm of *O*,*O*′‐Bis(2‐aminoethyl)ethylene glycol‐*N*,*N*,*N*′,*N*′‐tetraacetic acid (EGTA) was added to the standard bath solution without CaCl_2_. Fura2 fluorescence images were monitored at a 510‐nm emission with alternating excitation at 340 and 380 nm using Aquacosmos imaging system equipped with a cooled CCD camera (Hamamatsu Photonics, Hamamatsu, Japan). The ratio of the intensities excited at 340 and 380 nm was used to indicate the relative changes in intracellular Ca^2+^ concentration ([Ca^2+^]_*i*_). All measurements shown are representative of four independent experiments with no fewer than 10 cells.

### Statistical analysis

Data are presented as mean ± standard deviation (SD). Differences between groups were evaluated using one‐way analysis of variance (ANOVA) and two‐way ANOVA followed by Dunnett's or Tukey's test for multiple comparisons. Statistical analyses were performed using Prism 9 (GraphPad Software Inc., La Jolla, CA, USA). Statistical significance was defined by *P*‐value 0.05 [16].

## Results

### Effects of DEX on insulin secretion

**Fig. 1:**
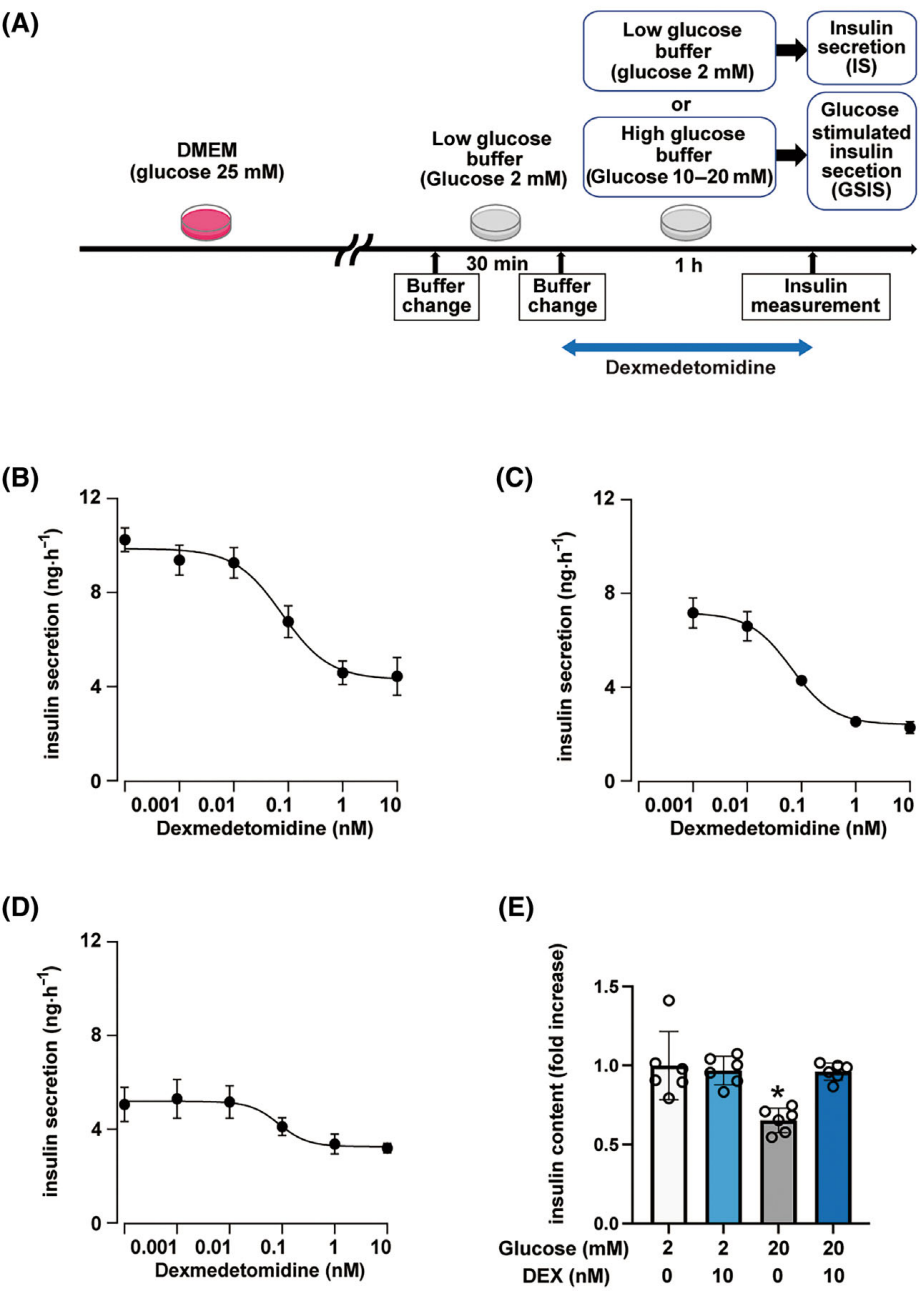
The effect of dexmedetomidine on insulin secretion in MIN‐6 cells. (A) Insulin secretion was induced by changing the glucose concentration of the buffer. (B) Mouse MIN6 cells were exposed to dexmedetomidine (DEX) for 1 h with 20 mm glucose. Insulin secretion was measured. Data are presented as mean ± SD (*n* = 6). The *solid line* is the fit by the Hill equation. Experiments were performed in triplicate. (C) Concentration‐response curve for DEX in 10 mm glucose (*n* = 6). (D) Concentration‐response curve for DEX in 2 mm glucose (*n* = 6). (E) Insulin content of cell lysate after insulin secretion experiments (*n* = 6). Differences between the treatment groups were evaluated using one‐way ANOVA followed by Dunnett's test for multiple comparisons. *P < 0.05, as compared with the control (glucose 2 mm) or the indicated group (glucose 20 mm, DEX 10 nm). ANOVA, analysis of variance; DEX, dexmedetomidine; GSIS, glucose‐stimulated insulin secretion; SD, standard deviation.

The influence of extracellular glucose concentration on insulin secretion was examined in mouse pancreatic β‐cell‐derived MIN6 cells. Insulin secretion was induced by changing the glucose concentration in the buffer, which was defined as insulin secretion (IS) and GSIS (Fig. 1A). DEX was maintained at a constant concentration until insulin secretion was measured. In previous experiments, we observed that GSIS increased in a glucose‐dependent manner and was saturated after approximately 1 h [6]. Thus, we incubated MIN6 cells with 0.1 pm to 10 nm DEX in 20 mm glucose for 1 h and measured the insulin concentrations. We observed that the GSIS significantly decreased in the group treated with 1 nm DEX compared with that of the control group in 20 mm glucose (Fig. 1B). The concentration‐response curve was fitted with a Hill equation and the half‐maximal inhibitory concentration (IC_50_) value was 0.079 ± 0.006 nm with a Hill coefficient of 0.935 ± 0.072 (*N* = 3). In 10 mm glucose, the IC_50_ value was 0.054 ± 0.012 nm with a Hill coefficient of 0.896 ± 0.226 (Fig. 1C; *N* = 2). In 2 mm glucose, the IC_50_ value was 0.040 ± 0.036 nm with a Hill coefficient of 1.341 ± 0.456 (Fig. 1D; *N* = 3). In addition, the insulin content of the cell lysate after insulin secretion experiments was not reduced by DEX treatment (Fig. 1E). The results indicated that clinically relevant doses of DEX have a suppressive effect on IS and GSIS.

### Effects of DEX on GSIS in rat INS‐1 cells and pancreatic β‐cell/islets

**Fig. 2:**
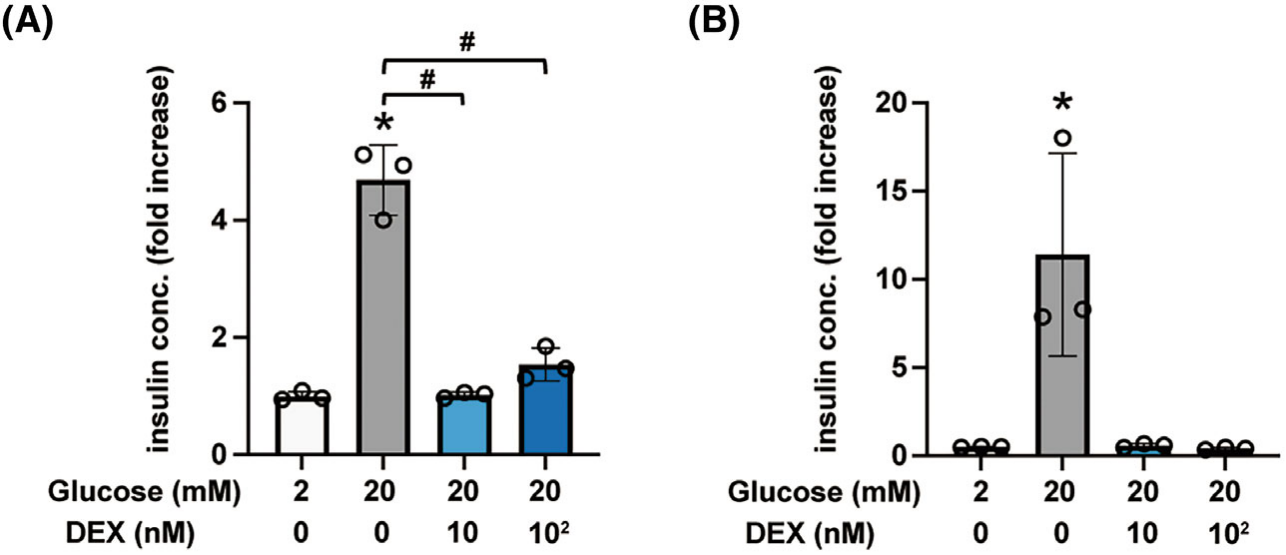
Effect of DEX on glucose‐stimulated insulin secretion in INS‐1 cells or mouse pancreatic β‐cells/islets. (A) INS‐1 cells were exposed to DEX (10 or 100 nm) for 1 h in 20 mm glucose. (B) Mouse pancreatic β‐cells/islets were exposed to DEX (10 or 100 nm) for 1 h in 20 mm glucose. In the case of β‐cells/islets, insulin concentrations were compensated with total protein weight. Insulin secretion was determined. Data are presented as mean ± SD (*n* = 3). Differences between treatments were evaluated using one‐way ANOVA followed by Dunnett's test for multiple comparisons. *P < 0.05, as compared with the control (glucose 2 mm); ^#^ *P* < 0.05 as compared with the indicated groups (glucose 20 mm, DEX 0 nm). ANOVA, analysis of variance; DEX, dexmedetomidine; SD, standard deviation.

The effects of DEX on GSIS were examined in rat pancreatic β‐cell‐derived INS‐1 cells and primary mouse pancreatic β‐cells/islets. DEX, at doses of 10–100 nm, significantly inhibited GSIS in the presence of 20 mm glucose for 1 h (Fig. 2A). β‐cells/islets were incubated with 10–100 nm DEX in 20 mm glucose for 1 h, and insulin concentration was assessed (Fig. 2B). In the case of β‐cells/islets, the insulin concentration was compensated by the total protein weight. Insulin secretion at a low‐glucose concentration was defined as 100% control and compared with other results. Similar to that in MIN6 and INS‐1 cells, 10 nm DEX suppressed GSIS after 1 h of incubation. Thus, DEX suppressed GSIS in the cell lines as well as in the primary mouse pancreatic β‐cells/islets.

### Effect of DEX on the death and energy metabolism of MIN6 cells

**Fig. 3:**
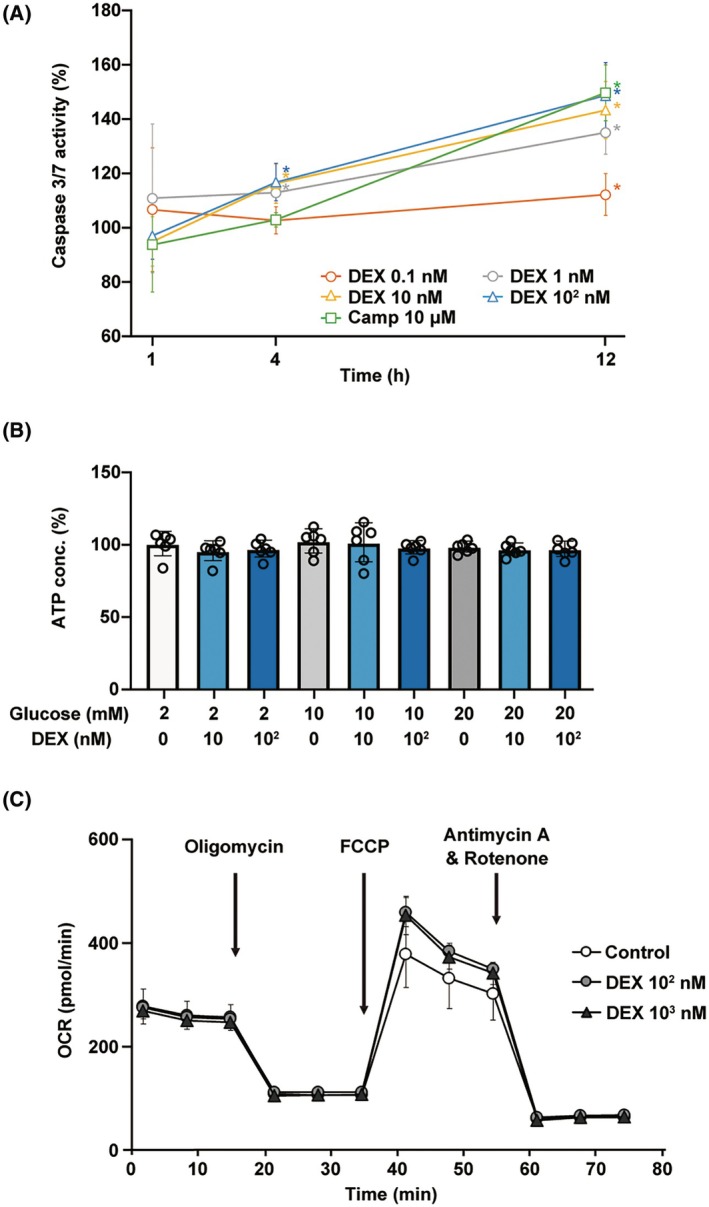
Effect of DEX on cell death and cellular energy metabolism of mouse MIN6 cells. (A) MIN6 cells were exposed to DEX at doses ranging from 0.1 to 100 nm and camptothecin at 10 μm and cultured for 1–12 h before determining cell viability by measuring caspase‐3/7 activity (*n* = 6). The percentage of caspase‐3/7 is expressed relative to the control (without DEX). (B) Mouse MIN6 cells were cultured for 1 h with DEX at doses of 10–100 nm before determining the cellular ATP level (*n* = 6) with 2–20 mm glucose. Experiments were performed in triplicates. (C) Mouse MIN6 cells were exposed to DEX at doses from 0 to 1 μm, followed by an OCR assay. Data are presented as mean ± SD (*n* = 3). Differences between treatments were evaluated using one‐way ANOVA followed by Dunnett's test for multiple comparisons. *P < 0.05, as compared with the control. ANOVA, analysis of variance; Camp, camptothecin; DEX, dexmedetomidine; OCR, oxygen consumption rate; SD, standard deviation.

The effect of DEX on cell death was investigated (Fig. 3A). DEX (0.1–100 nm) did not activate caspase‐3/7 within 1 h. However, DEX enhances caspase‐3/7 activity in a concentration‐ and time‐dependent manner after 4 h.

Intracellular ATP is generally assumed to play a crucial role in GSIS [11, 19], and high extracellular glucose levels increase ATP levels in pancreatic β‐cells [19]. The effect of DEX on ATP production in MIN6 cells was investigated after exposure to 2, 10, or 20 mm glucose for 1 h (Fig. 3B). We found that DEX did not affect ATP levels under any glucose concentration tested.

In our previous study, stimulation with high glucose levels activated mitochondrial respiration, and at 5 and 20 mm glucose, the OCR increased in a time‐dependent manner up to 4 h [6]. Although we investigated the effect of DEX on oxygen metabolism in MIN6 cells, 100 nm to 1 μm DEX did not significantly affect the OCR with 20 mm glucose treatment (Fig. 3C). Our results showed that DEX does not affect cell death, cellular oxygen, or energy metabolism of MIN6 cells within 1 h.

### Effects of DEX on insulin secretion enhanced by insulinotropic factors

**Fig. 4:**
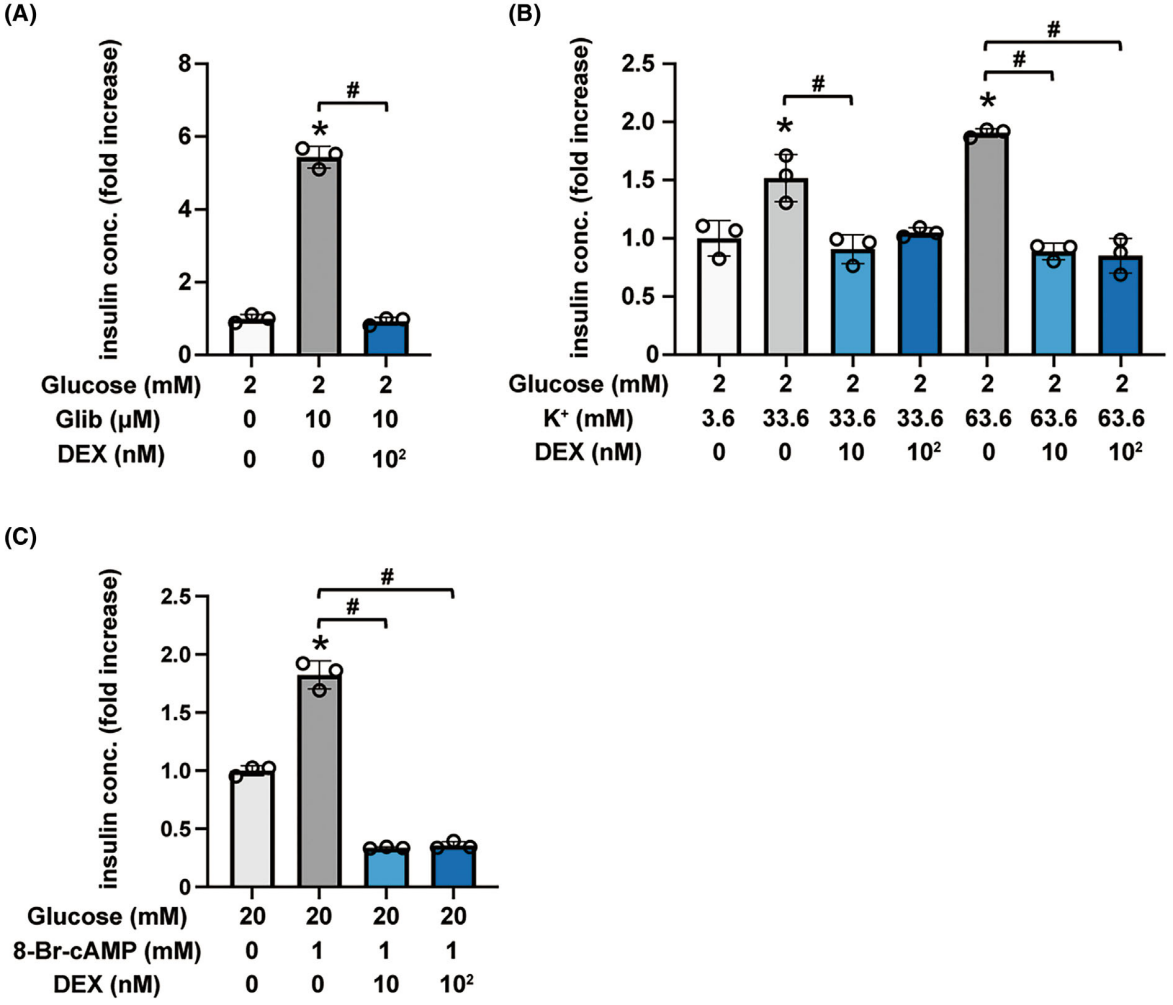
Effect of DEX on insulin secretion induced by glibenclamide, cAMP analogue, or potassium ion. (A) Mouse MIN6 cells were exposed to 100 nm DEX for 1 h with or without 10 μm glibenclamide in 2 mm glucose. (B) MIN6 cells were exposed to DEX (10 or 100 nm) for 1 h in KRBH buffer contained 3.6, 33.6, or 63.6 mm potassium ions. The osmolarity was not adjusted in this experiment. (C) Mouse MIN6 cells were exposed to DEX (10 or 100 nm) for 1 h with or without 1 mm 8‐Br‐cAMP in 20 mm glucose. Insulin secretion was determined. Data are presented as mean ± SD (*n* = 3). Differences between treatments were evaluated using one‐way ANOVA followed by Dunnett's test for multiple comparisons. *P < 0.05, as compared with the control (without glibenclamide, 8‐Br‐cAMP or potassium ion treatment); ^#^ *P* < 0.05 as compared with the indicated groups (DEX 0 μm). ANOVA, analysis of variance; DEX, dexmedetomidine; OCR, oxygen consumption rate; SD, standard deviation.

Glibenclamide, a K_ATP_ channel blocker, facilitates insulin secretion in pancreatic β‐cells even under low‐glucose conditions [19]. DEX treatment significantly suppressed insulin secretion induced by 10 μm glibenclamide compared with that of the control group without DEX or glibenclamide treatment (Fig. 4A). As it is well known, a high concentration of potassium ions facilitates Ca^2+^‐induced insulin secretion in pancreatic β‐cells even in low‐glucose conditions [20]. Compared to the control, 10 nm DEX inhibited Ca^2+^‐triggered insulin secretion potentiated by 33.6 or 63.6 mm potassium ions for 1 h (Fig. 4B).

To investigate whether cAMP elevation is involved in the DEX‐induced inhibition of insulin secretion, the effect of cAMP was examined. cAMP facilitates insulin secretion in pancreatic β‐cells under high glucose conditions [21]. We confirmed that the cAMP analog 8‐Br‐cAMP‐induced insulin secretion was significantly inhibited by 10 nm DEX (Fig. 4C).

### Effects of DEX on the ion channels in MIN6 cells

**Fig. 5:**
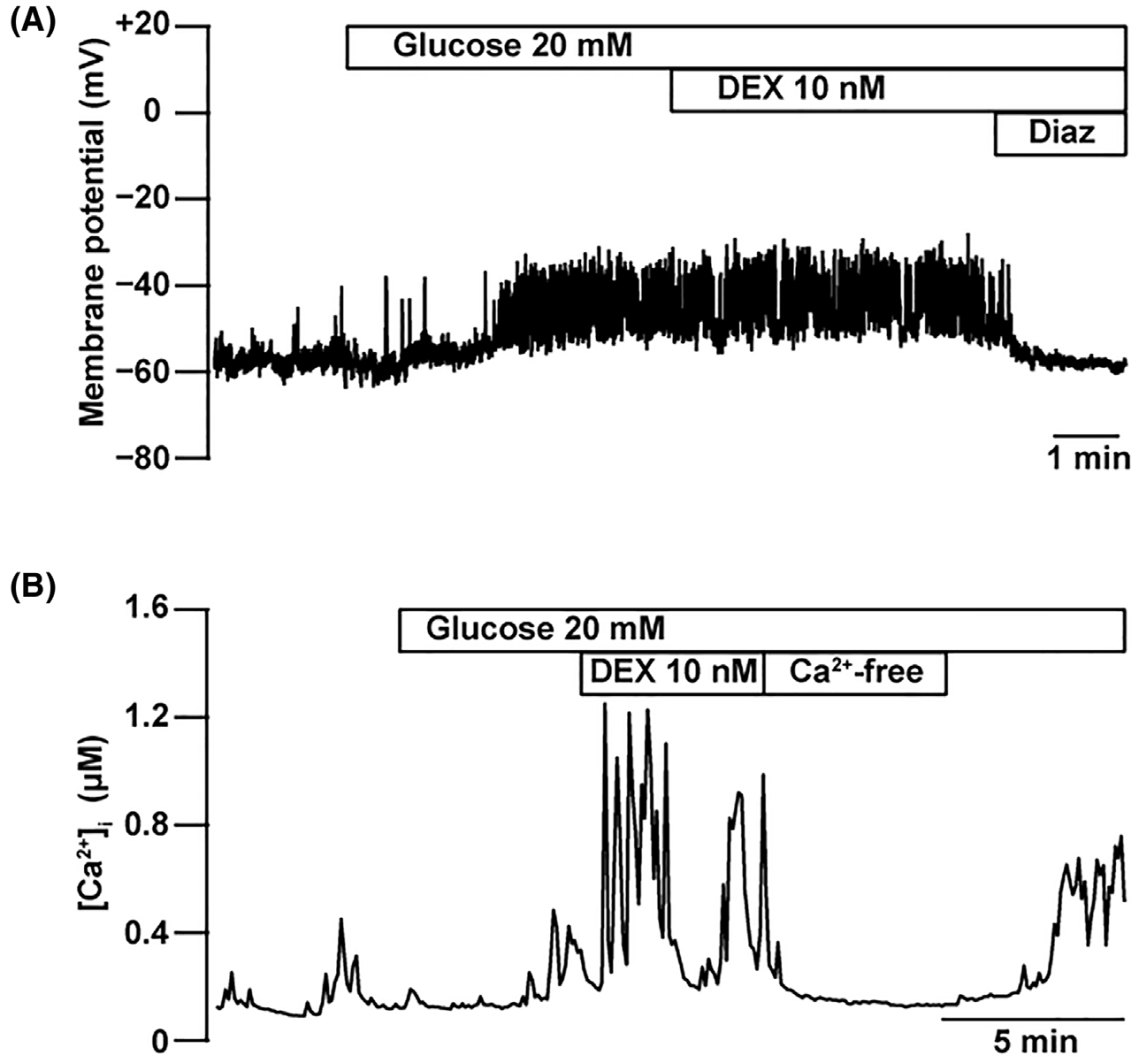
Effects of DEX on the membrane potential and intracellular Ca^2+^ concentration of mouse MIN6 cells. (A) Membrane potential was recorded using a gramicidin‐perforated patch. Glucose at a high concentration (20 mm) induced depolarization of the membrane potential. The addition of DEX (10 nm) had little effect on the membrane potential (*n* = 4). Diazoxide (Diaz, 100 μm) induced hyperpolarization, indicating an increase in K^+^ conductance through K_ATP_ channels. (B) The intracellular Ca^2+^ concentration ([Ca^2+^]_*i*_) was measured using the fluorescent calcium indicator dye fura 2‐AM. The addition of DEX (10 nm) did not inhibit the Ca^2+^ spike in 20 mm glucose, but subsequent perfusion of Ca^2+^‐free solution decreased [Ca^2+^]_*i*_ in MIN6 cells. Trace is representative of four independent experiments. DEX, dexmedetomidine.

Elevation of the intracellular ATP concentration in response to higher glucose conditions closes K_ATP_ channels and depolarizes the plasma membrane [17]. The membrane potential of MIN6 cells was measured using the gramicidin‐perforated patch technique [6, 18]. In this configuration, the intracellular glucose concentration was preserved because the area of the gramicidin‐perforated patch membrane was considerably smaller than that of the whole‐cell membrane. High glucose (20 mm) induced depolarization of the membrane potential (Fig. 5A). The application of DEX (10 nm) had little effect on the membrane potential (*n* = 4). However, the addition of a K_ATP_ channel opener (diazoxide, 100 μm) induced hyperpolarization, indicating an increase in K^+^ conductance in the plasma membrane of MIN6 cells. As it is well known, depolarization of the membrane potential increases intracellular Ca^2+^ concentration ([Ca^2+^]_*i*_) via voltage‐gated Ca^2+^ channels in pancreatic β‐cells [11]. The [Ca^2+^]_*i*_ was measured in single MIN6 cells using the fluorescent calcium indicator dye fura 2‐AM. The application of DEX (10 nm) did not inhibit the [Ca^2+^]_*i*_ in high glucose (Fig. 5B).

**Fig. 6:**
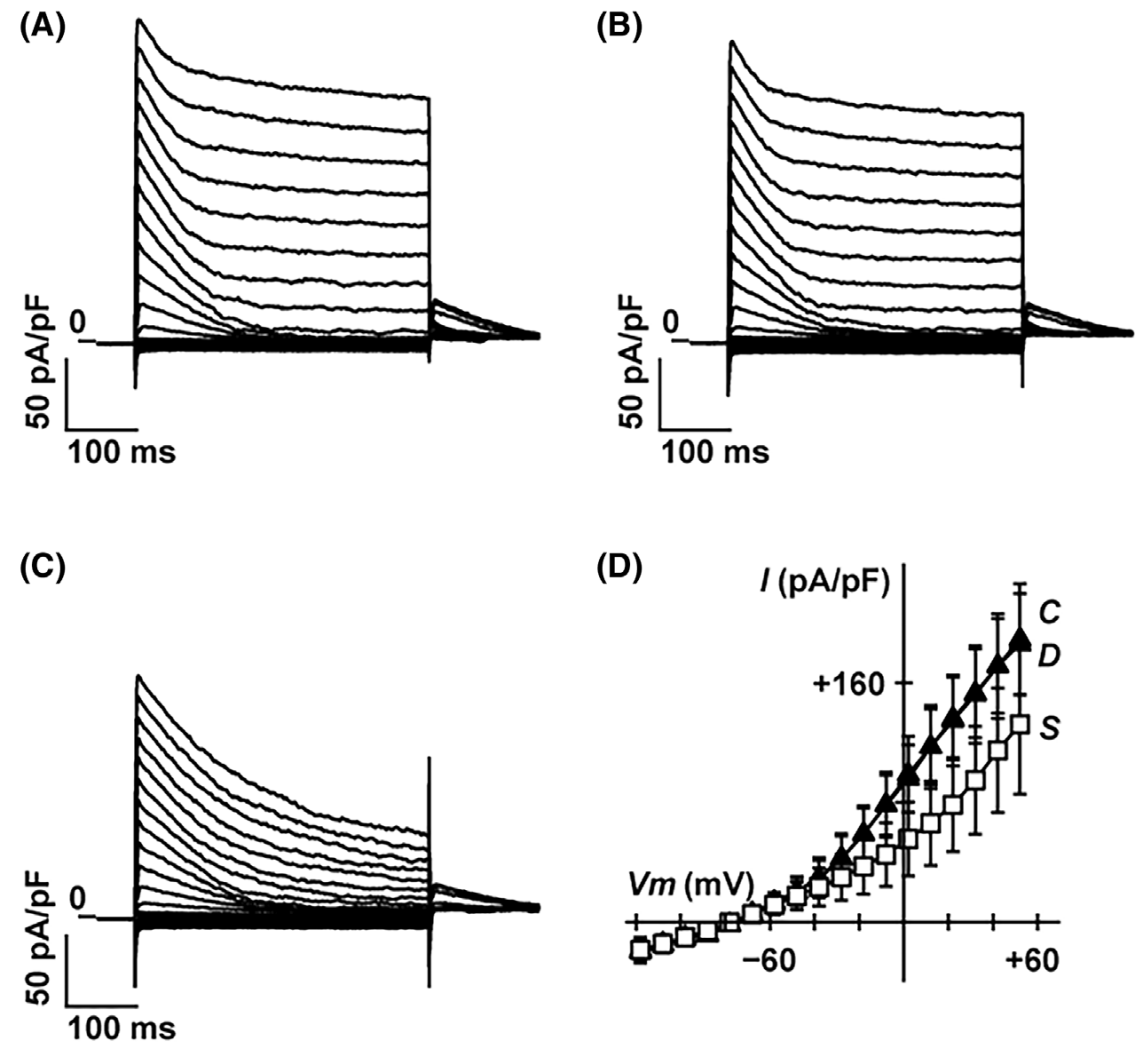
Effect of DEX on voltage‐dependent outward potassium currents in mouse MIN6 cells. Representative tracings of whole‐cell currents obtained from a mouse MIN6 cell in the absence (A) or presence of DEX (10 nm) (B) and stromatoxin‐1 (100 nm) (C). The cell was held at −88 mV and stepped up for 0.4 s to potentials ranging between −128 and +52 mV in 10 mV increments. (D) Current–voltage (*I–V*) relationships of whole‐cell currents in the steady state in the absence (*C*) or presence of DEX (*D*) and stromatoxin‐1 (*S*). DEX had little effect on outward potassium currents through the voltage‐dependent K^+^ channels. Data are presented as mean ± SD (*n* = 5). DEX, dexmedetomidine.

Previous studies have shown that the inhibition of voltage‐dependent K^+^ (K_v_) channels augments membrane depolarization and insulin secretion [6, 22, 23]. Whole‐cell currents were measured with a K^+^‐rich pipette solution containing 3 mm ATP using a voltage‐clamp configuration, and K_v_ currents were recorded in MIN6 cells bathed in 20 mm glucose buffer (Fig. 6A). The application of DEX (10 nm) had little effect on the K_v_ currents (Fig. 6B, *n* = 5). However, stromatoxin‐1 (100 nm), an inhibitor of the K_v_ 2.1/2.2 channels, inhibited both peak and sustained K_v_ currents (Fig. 6C) [6]. The current–voltage relationships of sustained K_v_ currents showed that DEX does not significantly affect outward potassium currents through K_v_ channels in MIN6 cells (Fig. 6D, *n* = 5).

**Fig. 7:**
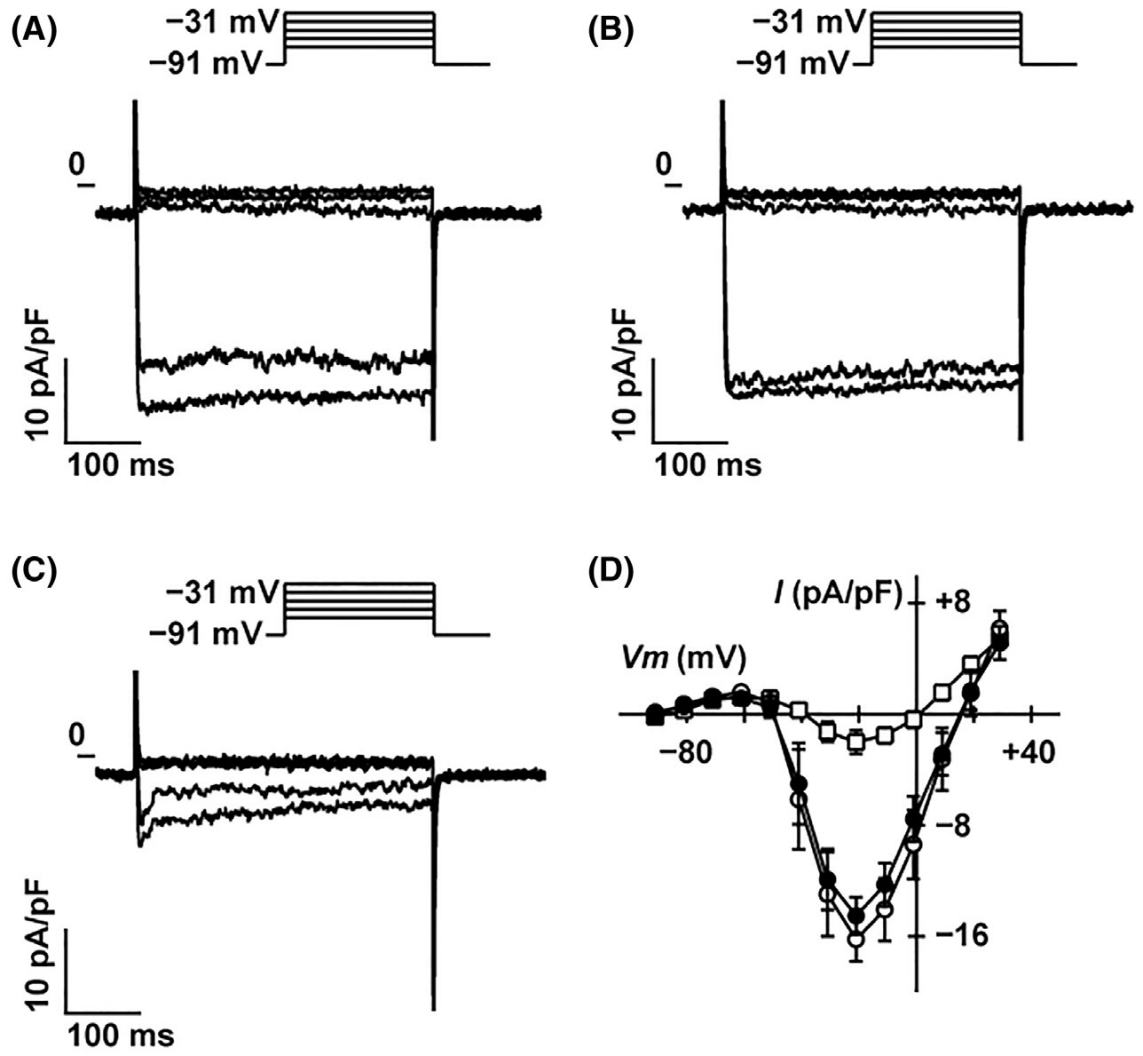
Effect of DEX on voltage‐dependent Ca^2+^ channels in mouse MIN6 cells. Representative tracings of whole‐cell currents obtained from a mouse MIN6 cell in the absence (A) or presence of DEX (10 nm) (B) and nifedipine (100 μm) (C). The cell was held at −91 mV and stepped up for 0.4 s to potentials ranging between −71 and −31 mV in 10 mV increments (inset). (D) Current–voltage (*I–V*) relationships of whole‐cell currents in the steady state in the absence (open circles) or presence of DEX (solid circles) and nifedipine (squares). DEX did not significantly affect inward barium currents through voltage‐dependent Ca^2+^ channels. Data are presented as mean ± SD (*n* = 6). DEX, dexmedetomidine.

A previous study demonstrated that glucose‐induced insulin secretion is almost completely abolished by pharmacological inhibitors of L‐type Ca^2+^ channels, such as nifedipine [24]. Whole‐cell currents were measured through voltage‐gated Ca^2+^ channels (VGCC) in MIN6 cells with a Cs^+^‐rich pipette solution (Fig. 7A). The application of DEX (10 nm) had little effect on inward currents (Fig. 7B, *n* = 6), which were decreased by nifedipine (Fig. 7C). The current–voltage relationship of sustained currents showed that DEX did not significantly affect inward currents through L‐type Ca^2+^ channels in MIN6 cells (Fig. 7D, *n* = 6). These results indicate that DEX had a negligible effect on K_ATP_, K_v_, and L‐type Ca^2+^ channels in MIN6 cells.

## Discussion

We demonstrated that the intravenous anesthetic DEX at clinically relevant doses significantly suppressed GSIS in MIN6 and INS‐1 cell lines as well as primary pancreatic β‐cells/islets. We examined several channels related to insulin secretion; however, we found that they were not affected by DEX.

In this study, DEX was used at concentrations ranging from 0.1 pm to 100 μm. The plasma concentrations of DEX, which is used clinically as an anesthetic and sedative, reportedly range between 0.27 (1 nm) and 1.37 ng·mL^−1^ (6 nm) [8]. A previous study has reported that the concentration of DEX in volunteers who were administered 2 μg·kg^−1^ DEX over 2 min could reach 2.3 ng·mL^−1^ (10 nm) [25]. The duration of DEX exposure in our study ranged from 1 to 12 h, which is also within the clinically used exposure period. Therefore, the concentrations of DEX and time range of its administration in this study are clinically relevant. We examined the effect of DEX on insulin secretion by measuring the amount of insulin released into buffer. If the insulin content of cells is considerably affected by DEX, it is possible that it will affect the insulin concentration released into the buffer. However, short‐term exposure to DEX did not sufficiently alter insulin content enough to explain the strong suppression observed in this study.

A convincing model of GSIS has been established based on considerable experimental evidence [11, 19, 26]. The extracellular glucose concentration stimulates pancreatic β‐cell metabolism leading to an increase in intracellular ATP concentration ([ATPi]) within β‐cells. The activity of the K_ATP_ channel decreases in response to increased [ATPi]. After that, the plasma membrane depolarizes at the threshold at which the VGCC opens. The influx of Ca^2+^ facilitates the exocytosis of insulin‐containing vesicles. Therefore, [ATPi] is generally assumed to play a crucial role in GSIS. In this study, DEX at concentrations less than 100 nm did not affect [ATPi], and the OCR was not affected by 1 μm DEX.

In the caspase experiment, we confirmed that DEX increased caspase‐3/7 activity in a concentration‐ and time‐dependent manner. However, caspase‐3/7 activation was observed only after 4 h and not at any concentration within 1 h. In this study, GSIS was significantly suppressed by DEX within 1 h. While prolonged DEX exposure may influence insulin secretion through apoptosis, the GSIS suppression observed at an early stage is likely independent of cellular damage. Notably, caspase activation was detected even at the clinically relevant concentration of 1 nm, making this as an important topic for future investigation.

Glibenclamide (K_ATP_ channel blocker) and 8‐Br‐cAMP (cAMP analogue) promote insulin secretion [19, 21]. However, we observed that DEX significantly inhibited insulin secretion induced by these drugs. Potassium ions at depolarizing concentrations in the cell membrane cause an influx of extracellular Ca^2+^, which elevates the free cytosolic Ca^2+^ concentration ([Ca^2+^]) and activates insulin secretion. In this study, DEX inhibited insulin secretion induced by potassium ions. Thus, DEX may inhibit GSIS by affecting insulin vesicle exocytosis.

In electrophysiological studies, we showed that DEX had no significant effect on the channels (K_ATP_ channel, K_v_ channel, and VGCC) involved in insulin secretion. Kodera *et al*. [9] indicated that DEX inhibits insulin secretion through the α2‐adrenoceptor and PTX‐sensitive GTP‐binding protein pathways, which involve K_v_ channel activation and Ca^2+^ channel inhibition. However, they also proposed that DEX inhibits insulin secretion via pathways other than that involved in the mechanism proposed above. In the present study, we did not observe K_v_ channel activation or Ca^2+^ channel inhibition by DEX. Moreover, the intracellular Ca^2+^ concentration was not inhibited by DEX. This discrepancy in the results might be partially explained by differences in the cell lines or culture conditions used. It is well known that adrenaline suppresses insulin secretion [27]. Therefore, it is possible that the mechanism of inhibition of insulin secretion by adrenaline and DEX might be similar. However, it does not ensure that the inhibitory mechanism of DEX is identical to that of adrenaline because DEX and adrenaline are drugs that have different structures. In addition to the findings of a previous study [9], our study suggests that insulin secretion is mainly inhibited by another pathway.

DEX contains an imidazole ring in its structure and interacts with imidazoline receptors [28]. Some imidazoline derivatives have been shown to increase insulin response to glucose [29]. A previous study showed that this insulinotropic effect is not observed at low clinical DEX concentrations [9]. The DEX‐induced increase in insulin secretion via imidazoline receptors may be a minor effect compared to the DEX‐induced inhibition of insulin secretion via α2‐adrenoceptors. Takahashi *et al*. [8] also reported that DEX has a dual action on insulin secretion: predominant inhibition via the α2‐adrenoceptor and weak stimulation via the imidazoline receptor. In their study, DEX at above‐normal sedative doses affected insulin secretion. However, we observed the inhibition of insulin secretion at clinically relevant doses. The above results indicate that DEX inhibits GSIS via α2‐adrenoceptor or by insulin vesicle exocytosis.

This study had several limitations. First, we did not directly observe the inhibition of insulin vesicle exocytosis; it is unclear whether DEX inhibits specific steps of a K_ATP_ channel‐independent pathway or insulin vesicle exocytosis. The fusion of transport vesicles with their target membranes is driven by the assembly of functional soluble N‐ethylmaleimide‐sensitive factor attachment protein receptor (SNARE) complexes [30]. The v‐SNAREs (such as VAMP2 and VAMP8) and t‐SNAREs (such as syntaxin 1A and syntaxin 3) are SNARE proteins that reportedly localize to insulin granules and pancreatic β‐cell membranes and promote insulin secretion [31, 32, 33]. To elucidate the precise mechanism, we will observe the insulin vesicle in real time using fluorescence microscopy or study the effect of the knockout of genes encoding these proteins in our future work. Moreover, Henquin *et al*. [34, 35] have shown insulin secretion mechanisms independent of K_ATP_ channels, and it may be possible to use their methods to reveal more detailed mechanisms. Second, we only examined tumor‐derived mouse MIN6 cells, rat INS‐1 cells, and mouse pancreatic islets; therefore, further studies on cells or islets of human origin are warranted to validate the clinical application of these findings. We performed electrophysiological studies exclusively using MIN6 cells. MIN6 cells are insulinoma cell lines derived from a transgenic mouse expressing the large T‐antigen SV40 in pancreatic β‐cells [10]. Several studies have indicated that the characteristics of MIN6 cells are similar to those of isolated islets, indicating that this cell line is an appropriate model for studying the mechanism of GSIS in pancreatic β‐cells [10, 36, 37]. In this study, we did not perform *in vivo* experiments because we focused on the mechanism of action of DEX in insulin secretion. Although substantial hyperglycemia in patients treated with DEX is uncommon in clinical practice, it could be caused by stress or pharmacological combination with other medications. According to Uyar *et al*. [37], DEX may reduce the sympathoadrenal response to stress and substantially limit the hyperglycemic response. Further *in vivo* experiments are warranted to determine the effect of DEX on systemic glucose metabolism.

## Conclusions

In conclusion, DEX, at clinically relevant doses, potentially inhibits a signaling pathway via α2‐adrenoceptor or insulin vesicle exocytosis to suppress GSIS in pancreatic β‐cells. DEX does not induce cytotoxicity and does not affect channels involved in insulin secretion. Our results support the hypothesis that DEX suppresses insulin secretion and reveal the underlying mechanisms.

## Conflict of interest

The authors declare that they have no conflict of interest.

### Peer review

The peer review history for this article is available at https://www.webofscience.com/api/gateway/wos/peer‐review/10.1002/2211‐5463.13960.

## Author contributions

MK, MH, YM, and KH conceived and designed the experiments. MK and MH acquired and analyzed the experimental data. MK, MH, and KH prepared the figs. MH, YM, and KH provided experimental materials. All authors read and approved the final version of the manuscript.

## Data Availability

The datasets used and/or analyzed during the current study are available from the corresponding author on reasonable request.
